# Decanoic Acid Exerts Its Anti-Tumor Effects via Targeting c-Met Signaling Cascades in Hepatocellular Carcinoma Model

**DOI:** 10.3390/cancers15194681

**Published:** 2023-09-22

**Authors:** Min Hee Yang, Mina Lee, Amudha Deivasigamani, Duc Dat Le, Chakrabhavi Dhananjaya Mohan, Kam Man Hui, Gautam Sethi, Kwang Seok Ahn

**Affiliations:** 1 Department of Science in Korean Medicine, Kyung Hee University, Seoul 02447, Republic of Korea; didmini@naver.com; 2 College of Pharmacy, Sunchon National University, 255 Jungangno, Suncheon-si 57922, Republic of Korea; minalee@scnu.ac.kr (M.L.); ddle@scnu.ac.kr (D.D.L.); 3 Division of Cellular and Molecular Research, Humphrey Oei Institute of Cancer Research, National Cancer Centre Singapore, Singapore 169610, Singapore; amudha.deivasigamani@nccs.com.sg (A.D.); cmrhkm@nccs.com.sg (K.M.H.); 4 FEST Division, CSIR-Indian Institute of Toxicology Research, Vishvigyan Bhawan, 31, Mahatma Gandhi Marg, Lucknow 226001, Uttar Pradesh, India; cd.mohan@yahoo.com; 5 Department of Pharmacology, Yong Loo Lin School of Medicine, National University of Singapore, Singapore 117600, Singapore

**Keywords:** capric acid, c-Met, hepatocellular carcinoma, orthotopic mouse model

## Abstract

**Simple Summary:**

Hepatocellular carcinoma (HCC) is a primary liver malignancy that remains a fatal disease with limited therapeutic options. Aberrant activation of c-Met can modulate tumor growth and progression in HCC. Herein, we have examined the anti-neoplastic effects of decanoic acid (DA) in hepatocellular carcinoma cells and in vivo mouse models. DA suppressed the phosphorylation of c-Met and induced apoptosis in HCC cells by inhibiting the expression of various oncogenic proteins. Moreover, DA inhibited the c-Met cascade in the preclinical cancer model. These results support the idea that DA can be considered a new anti-tumor agent for HCC.

**Abstract:**

DA, one of the medium-chain fatty acids found in coconut oil, is suggested to have diverse biochemical functions. However, its possible role as a chemoprevention agent in HCC has not been deciphered. Aberrant activation of c-Met can modulate tumor growth and progression in HCC. Here, we report that DA exhibited pro-found anti-tumor effects on human HCC through the suppression of HGF/c-Met signaling cascades in vitro and in vivo. It was noted that DA inhibited HGF-induced activation of c-Met and its downstream signals. DA induced apoptotic cell death and inhibited the expression of diverse tumorigenic proteins. In addition, DA attenuated tumor growth and lung metastasis in the HCC mouse model. Similar to in vitro studies, DA also suppressed the expression of c-Met and its downstream signals in mice tissues. These results highlight the substantial potential of DA in the prevention and treatment of HCC.

## 1. Introduction

Hepatocellular carcinoma (HCC) is a lethal disease that is often diagnosed in the advanced stages, which results in poor overall survival and a dismal prognosis. As there are no specific symptoms in the early stages of the disease, most HCC patients are generally diagnosed with an advanced stage [1,2,3]. It is the fourth leading cause of cancer-related death in the world [4,5]. Tyrosine kinase inhibitors, or immune checkpoint blockers, are used to treat patients with advanced HCC. However, patient responses to these therapies are fairly limited [6,7,8]. Therefore, there is an urgent need to develop novel therapeutic targets against this malignancy.

c-Met is a receptor tyrosine kinase that is stimulated by its cognate ligand, hepatocyte growth factor (HGF) [9]. In a conventional signaling pathway, HGF acts on the c-Met receptor in an autocrine or paracrine fashion. The interaction of HGF with c-Met results in the receptor homodimerization and autophosphorylation of a series of tyrosine residues located on the cytoplasmic domains of c-Met. These phosphorylation marks serve as a docking site for phospho-tyrosine-binding proteins such as SHIP2, PI3K, GRB2, and GAB1, which results in the activation of a broad range of signaling pathways such as the MAPK axis, the PI3K/Akt/mTOR axis, the STAT3 pathway, and the NF-κB pathway, leading to tumorigenesis [10]. Activation of these pathways promotes several processes in tumor cells, such as promoting proliferation, epithelial-mesenchymal transition (EMT), migration, angiogenesis, and antiapoptosis [10,11]. Overactivation or upregulated expression of c-Met has been witnessed in many types of human malignancies and is associated with poor clinical outcomes in patients with various carcinomas, including HCC [12,13,14,15]. Interestingly, deregulated expression of c-Met has been linked with both metastasis and a poor prognosis in HCC patients. Therefore, c-Met can be considered a useful therapeutic target for HCC therapy [16,17,18]. We have previously demonstrated that c-Met inhibition by natural compounds can suppress the growth of HCC cells [19]. Considering the importance of c-Met inhibition in cancer therapeutics, many natural/synthetic agents have been tested for their inhibitory efficacy against c-Met in a preclinical setting [20]. It was recently shown that Halorotetin A, a terpenoid isolated from *Halocynthia roretzi*, downmodulated the expression of c-Met and reduced the proliferation of HCC cells [21]. The use of DE605 (a c-Met inhibitor) in conjunction with sorafenib significantly suppressed HCC proliferation and metastasis in preclinical studies [22]. Curcumin impeded EMT in meningioma cells by targeting the HGF/c-Met axis and consequently abrogating the PI3K/Akt/mTOR pathway [23]. Resveratrol attenuated the HGF-mediated crosstalk between stroma and epithelium and reduced prostate cancer cell migration by suppressing the EMT process [24]. Butein was found to inhibit the growth of gefitinib-sensitive as well as gefitinib-resistant cells by mitigating the kinase activity of EGFR and c-Met in lung cancer cells. These studies suggest that targeting the c-Met pathway could be a good strategy for combating cancer cell proliferation, migration, EMT, metastasis, and drug resistance.

In this line, we have examined the effect of decanoic acid (DA), also known as capric acid, a saturated ten-carbon medium-chain fatty acid, on the c-Met signaling pathway in HCC cells [25]. DA is present in a wide range of natural substances, such as palm oil, coconut oil, milk fat, and parmesan cheese [25]. DA has been reported to possess pharmacological properties such as anti-fungal, anti-inflammatory, and anti-viral activities [26,27]. Also, DA, as well as a few other medium-chain fatty acids, have demonstrated good cytotoxic effects on cancers of the colorectal, skin, and breast [28,29]. However, the precise molecular mechanism of DA-induced cytotoxicity in HCC has not been clearly understood. Herein, we have examined the effect of DA on HCC cell lines and mouse models. We noted that DA reduced tumor growth and induced apoptosis by modulating the c-Met signaling pathway. 

## 2. Results

### 2.1. DA Caused Loss of Viability in HCC Cells

**Figure 1:**
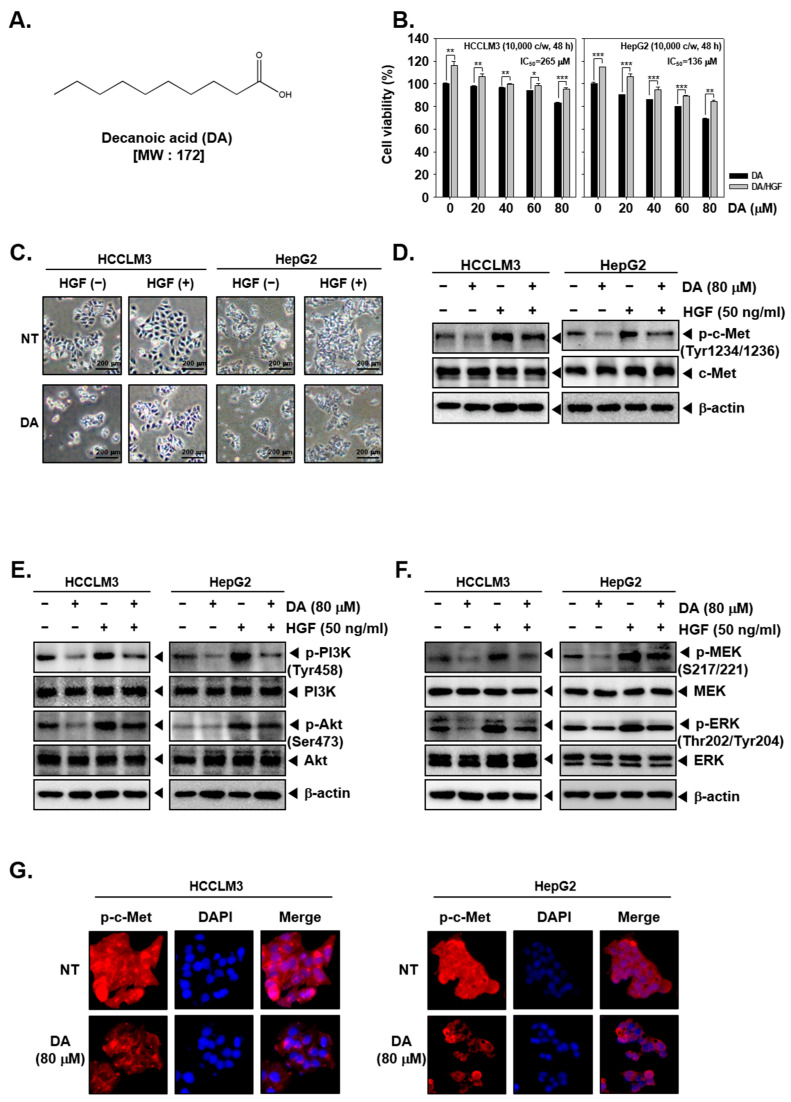
DA suppressed HGF---induced c-Met activation. (**A**) The chemical structure of decanoic acid (DA). (**B**) HCCLM3 and HepG2 cells were pre-treated with DA (0, 20, 40, 60, and 80 μM) for 2 h, then treated with 50 ng/mL of HGF for a total of 48 h. A 2,5-diphenyl-2H-tetrazolium bromide (MTT) assay was carried out to determine cell viability. * *p*, ** *p*, *** *p* < 0.05, 0.01, 0.001, compared to the control, respectively. (**C**) The cells were pre-treated with DA (80 μM) for 2 h and treated with HGF (50 ng/mL) for a total of 24 h. Morphological changes in cells were observed. (**D**–**F**) The cells were pre-treated with DA (80 μM) for 2 h and then treated with 50 ng/mL of HGF for a total of 30 min. Then, western blot analysis was performed. (**G**) HCCLM3 and HepG2 cells were treated with DA, and the expression of p-c-Met was evaluated by immunocytochemistry. The uncropped blots are shown in File S1.

The structure of DA has been depicted in Figure 1A. First, the effect of DA on the viability of HCCLM3 and HepG2 cells was examined. The cells were pre-treated with different doses of DA (0, 20, 40, 60, and 80 μM) for 2 h and then exposed to HGF (50 ng/mL) for a total of 48 h. There was a gradual loss of cell viability upon DA exposure in a dose-dependent manner (Figure 1B). Also, HGF-treatment increased the number of viable cells, and the pre-treatment of HCC cells with different doses of DA significantly curbed the HGF-driven cell proliferation, indicating that DA can reduce the cell viability in HGF-induced/uninduced HCC cells (Figure 1B). As shown in Figure 1C, HGF caused morphological alterations, which were reversed by DA. 

### 2.2. DA Suppressed the Constitutive/HGF-Induced Activation of c-Met in HCC Cells

Next, the impact of DA on constitutive/HGF-induced activation of c-Met and PI3K, Akt, MEK, and ERK was examined using Western blotting and immunocytochemistry. As shown in Figure 1D, there was a constitutive phosphorylation of c-Met in both HCC cells, whereas HGF treatment resulted in a multifold increase in the c-Met phosphorylation. The treatment of HCC cells with DA suppressed constitutive as well as HGF-induced c-Met phosphorylation. In addition, the effect of DA on constitutive/HGF-induced activation of oncogenic axes such as PI3K/Akt/mTOR and MEK/ERK. DA imparted marked inhibitory efficacy on constitutive/HGF-induced phosphorylation of all the tested proteins (Figure 1E,F). Moreover, immunocytochemistry experiments also revealed that DA suppresses the expression of phosphorylated-c-Met induced by HGF (Figure 1G).

### 2.3. DA Promoted Apoptosis in HCC Cells

**Figure 2:**
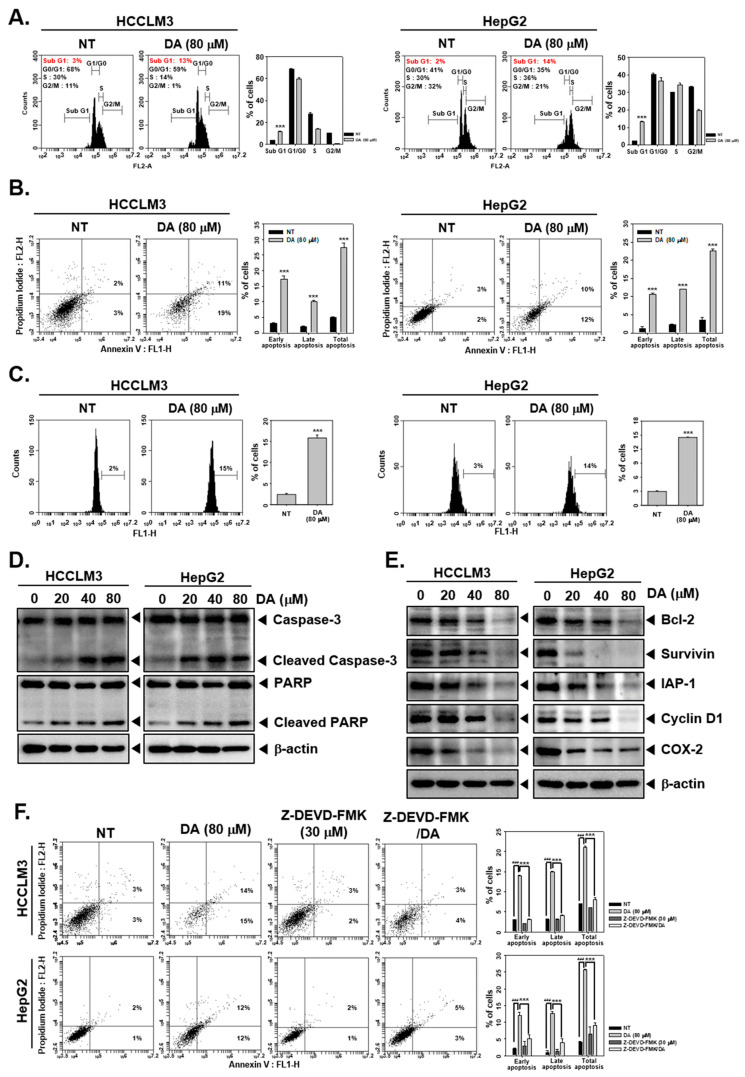
DA promotes apoptosis. (**A**–**C**) HCCLM3 and HepG2 cells were treated with DA (80 μM) for 24 h, and cell cycle analysis, annexin V, and TUNEL assays were performed. (**D**,**E**) Cells were incubated with various concentration conditions for 24 h, and immunoblotting for various proteins was carried out. *** *p* < 0.001, compared to the control. (**F**) The cells were treated with DA (80 μM) and Z-DEVD-FMK (30 μM) for 24 h, an annexin V assay was done. ^###^ *p* < 0.001, compared to the non-treated group. *** *p* < 0.001, compared to the DA-treated group. The uncropped blots are shown in File S1.

The effect of DA on the cell cycle distribution of HCCLM3 and HepG2 cells was investigated using a flow cytometer. HCCLM3 and HepG2 cells were exposed to DA (80 μM) for 24 h and stained with PI, followed by an analysis of the cell cycle pattern. DA increased the percentage of cells in subG1, indicating that cells are driven towards death (Figure 2A). Further, an annexin-V assay and a TUNEL assay were performed to verify whether DA imparts cell death through apoptosis. The results of both assay systems confirmed that DA induces apoptosis in tested cells (Figure 2B,C). There was an increase in annexin-V^+^ and PI^+^ cells upon exposure to DA, indicating the presence of early and late-apoptotic cells. Western blotting analysis was performed to determine the expression of apoptosis-related proteins in untreated and DA-treated cells. DA was found to induce the activation of caspase-3 and PARP (Figure 2D). Additionally, antiapoptotic proteins such as Bcl-2, Survivin, IAP-1, Cyclin D1, and COX-2 were found to be downregulated in DA-treated cells in comparison with untreated cells (Figure 2E). In an interesting observation, exposure of HCCLM3 and HepG2 cells to Z-DEVD-FMK, an inhibitor of caspase-3, resulted in inhibition of the apoptosis-inducing effect of DA and increased the number of viable cells, confirming that DA induces the apoptosis form of cell death (Figure 2F).

### 2.4. DA Stimulated Apoptosis in HGF-Induced Cells

**Figure 3:**
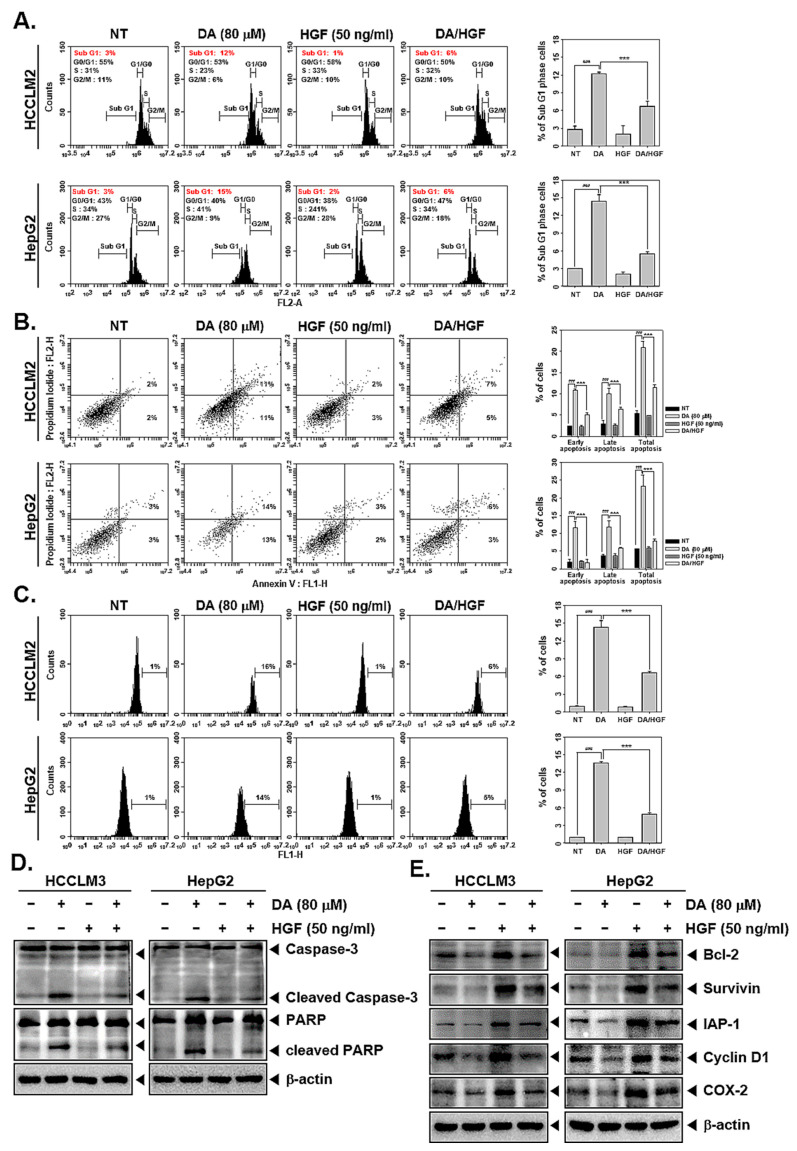
DA stimulated apoptosis in HGF-induced cells. HCCLM3 and HepG2 cells were exposed to DA (80 μM) and then treated with HGF for a total of 24 h. (**A**) The cells were incubated with RNase A, and cell cycle analysis was performed by flow cytometer. ^###^ *p* < 0.001, compared to the non-treated group. *** *p* < 0.001, compared to the DA-treated group. (**B**) The annexin V assay for determining apoptosis was carried out. ^###^ *p* < 0.001, compared to the non-treated group. *** *p* < 0.001, compared to the DA-treated group. (**C**) The cells were fixed with 4% paraformaldehyde and then stained with TUNEL assay reagent. ^###^ *p* < 0.001, compared to the non-treated group. *** *p* < 0.001, compared to the DA-treated group. (**D**,**E**) Western blot was performed to measure levels of different proteins. The uncropped blots are shown in File S1.

Further, the effect of DA on the survival of HGF-stimulated cells was investigated. HCCLM3 and HepG2 cells were pre-treated with DA (80 μM) and then exposed to HGF. These cells were used to perform PI staining, an annexin-V assay, a TUNEL assay, and western blotting. PI staining revealed that DA increases the sub-G1 cell population in both HGF-induced/uninduced cells, indicating cell death (Figure 3A). The results of the annexin-V assay and the TUNEL assay confirmed that DA triggers apoptosis in HGF-induced/uninduced cells (Figure 3B,C). Western blotting profiling of apoptosis-related proteins such as caspase-3, PARP, Bcl-2, Survivin, IAP-1, Cyclin D1, and COX-2 confirmed the apoptosis-inducing effect of DA in HGF-induced cells (Figure 3D,E).

### 2.5. DA Displayed No Significant Toxicity in NCr Nude Mice

**Figure 4:**
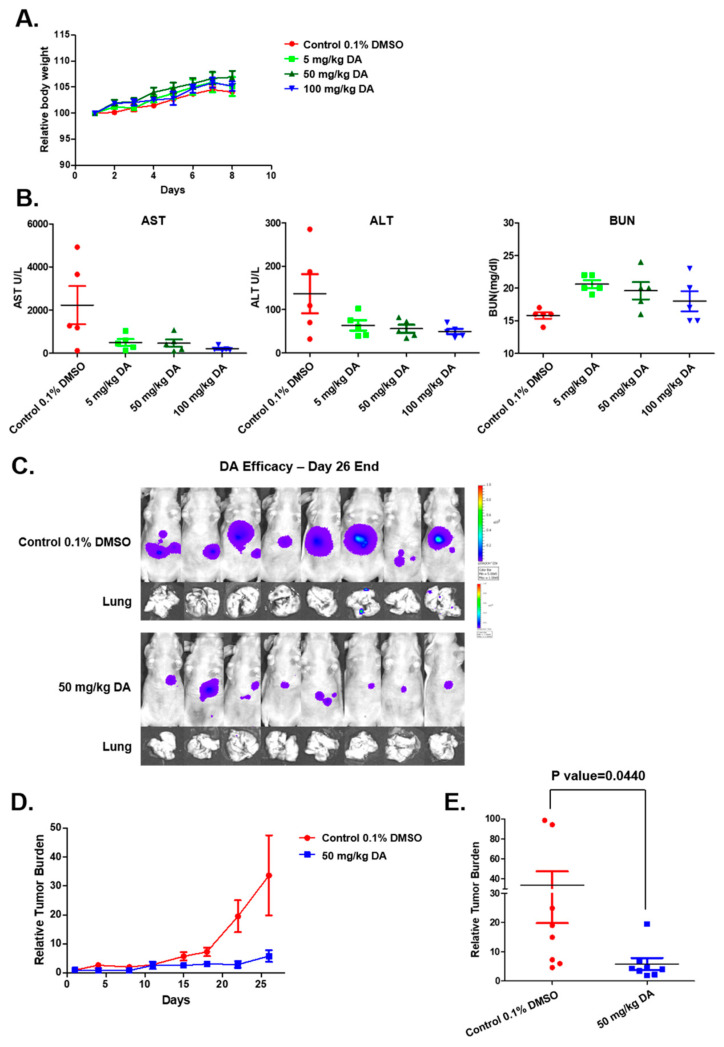
Effect of DA in the mouse model. (**A**) The graph depicts the body weight of mice. (**B**) The activity of AST, ALT, and BUN to study the toxicity profile of DA. (**C**) Growth-inhibitory effect of DA on HCCLN3-Luc cells implanted in mice. (**D**,**E**) Tumor burden was determined in mice.

An acute toxicity study was performed using eight-week-old NCr-nude female mice. For this, mice were intraperitoneally injected with indicated doses of DA (5, 50, and 100 mg/kg) and vehicle (0.1% DMSO). DA treatment caused no substantial effects on body weight (Figure 4A) or on the levels of various enzymes in the liver and kidney (Figure 4B).

### 2.6. DA-Attenuated Tumorigenesis in an Orthotopic Mice Model

The anti-tumor activity of DA was investigated in the HCC mouse model. Tumors derived from HCC-Luc cells were implanted into NCr-nude mice and allowed to form the tumor. Subsequently, tumor-bearing animals were intraperitoneally administered with either vehicle alone or DA alone (50 mg/kg). There was significant regression in the growth of tumors in DA-treated animals, with a reduction in lung metastasis (Figure 4C–E). Overall, DA treatment could effectively mitigate tumorigenesis without displaying toxicity in experimental animals.

### 2.7. DA-Suppressed Activation of c-Met and Downstream Molecules in Tumor Tissues

**Figure 5:**
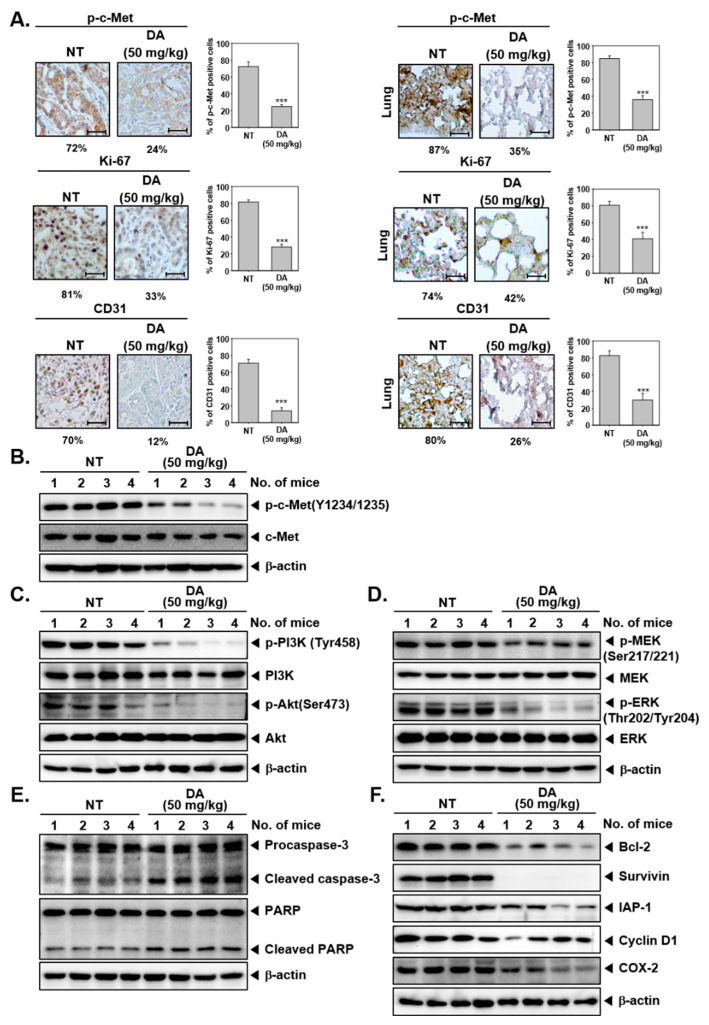
Impact of DA on c-Met on oncogenic proteins in the HCC mouse model. (**A**) p-c-Met, Ki-67, and CD31 were evaluated by immunohistochemistry. Quantification has been shown as mean ± SD on the right panel (scale bar: 200×). Data represents mean ± SD. *** *p* < 0.001, compared to control. (**B**–**F**) The expression of c-Met-dependent signaling cascades and apoptotic markers was determined using western blotting in the mouse tumor tissues. The uncropped blots are shown in File S1.

Further, the expression of p-c-Met, Ki-67, and CD31 was determined in the tumor tissues by immunohistochemical analysis. As displayed in Figure 5A, expression of p-c-Met, Ki-67, and CD31 was significantly reduced in tissue samples derived from the DA-treated group in comparison with the tissue samples taken from control animals (Figure 5A). As evidenced by the results of western blotting of tumor tissue samples, DA markedly abrogated activation of c-Met, PI3K, Akt, mTOR, MEK, and ERK (Figure 5B–D). DA was found to significantly increase the activation of PARP and caspase-3 as well as reduce the levels of antiapoptotic proteins such as Bcl-2, Survivin, IAP-1, Cyclin D1, and COX-2 (Figure 5E,F).

## 3. Discussion

Since the discovery of c-Met and its high-affinity ligand, HGF, numerous efforts have been made to understand the role of the HGF/c-Met axis in carcinogenesis. The HGF/c-Met axis plays a crucial role in embryogenesis and early developmental stages. It also modulates cell proliferation, EMT, immune response, morphogenesis, apoptosis, and angiogenesis. All the studies reported to date have emphasized the prominent oncogenic role of the HGF/c-Met axis in human cancers. Some studies have demonstrated a multifold increase in c-Met expression in HCC tissues compared to normal liver tissues without the corresponding increase in HGF, indicating the independent role of c-Met in hepatocarcinogenesis [30,31,32,33,34,35]. Many c-Met inhibitors, such as Glumetinib, Crizotinib, Tepotinib, ABN401, Tivantinib, and many more, are being examined in clinical trials against various human malignancies. The HGF/c-Met axis is also shown to promote resistance against chemotherapeutic agents. Sorafenib-resistant HCC cells were demonstrated to secrete HGF to activate c-Met receptors in an autocrine fashion, which ultimately results in sustained oncogenic signals, and these cells displayed elevated expression of mesenchymal proteins with enhanced invasive ability [36]. Mutations and other aberrations in the c-Met gene, including activating mutations, splicing mutations, amplifications, and rearrangements, are reported to be associated with the dysregulation of the c-Met signaling cascade. Activating mutations in the tyrosine kinase domain (such as M1149T, V1206L, L1213V, V1238I, D1246N, D1246H, Y1248C, Y1248H, and M1268T) were detected in human cancers [37,38]. Accumulating evidence has suggested that inhibition of HGF/c-Met signaling could serve as a useful therapeutic strategy for attenuating the growth and metastasis of various human cancers, including HCC, colorectal cancer, lung cancer, head and neck cancer, cervical cancer, etc. [39]. Numerous studies are underway in preclinical and clinical settings to discover new inhibitors of the c-Met pathway to counteract the growth of c-Met-positive tumors. Thus, inhibition of c-Met and subsequently its dependent kinases can be a potential therapeutic target to develop novel pharmacological agents against a variety of malignancies [19,40,41,42,43]. We and others have also previously reported the inhibitory activity of some natural/synthetic compounds against the c-Met pathway in preclinical cancer models. For instance, a previous report demonstrated that fangchinoline can exhibit anti-neoplastic actions through attenuation of the c-Met pathway [19]. Ginkgolide C was also able to suppress the activation of c-Met and its downstream cascades [40]. Foretinib, an inhibitor of c-Met and multiple kinases, reduced tumor growth in patient-derived HCC xenograft models [44]. Apigenin, artonin F, carnosol, celastrol, curcumin, damnacanthal, deguelin, epigallocatechin-3-gallate, evodiamine, licochalcone A, luteolin, α-mangostin, meleagrin, oleocanthal, osthole, quercetin, quinacrine, resveratrol, rutin, salvianolic acid A, verticillin A, and withaferin A are some of the other natural products with c-Met inhibitory activity. Some fatty acids, including DA, have been demonstrated to possess some pharmacological activities in different disease models. Lauric acid was found to downregulate the EGFR signaling pathway in colon cancer cells to impart cell death [45]. Lauric acid also imparted apoptosis in endometrial and breast cancer cells [46]. Thakur and colleagues recently reviewed the potential effects of DA on neurological disorders [47]. DA was reported to curb the expression of inflammatory and metastasis markers in colon cancer cells [28]. No reports have precisely indicated the mechanism by which DA imparts activity in the HCC model. In continuation of our efforts in the exploration of new inhibitors of oncogenic proteins, we have investigated the effect of DA on the c-Met and its dependent pathways in HCC. We demonstrated that DA exerted anti-cancer activities by targeting HGF/c-Met signaling in the HCC model. 

**Figure 6:**
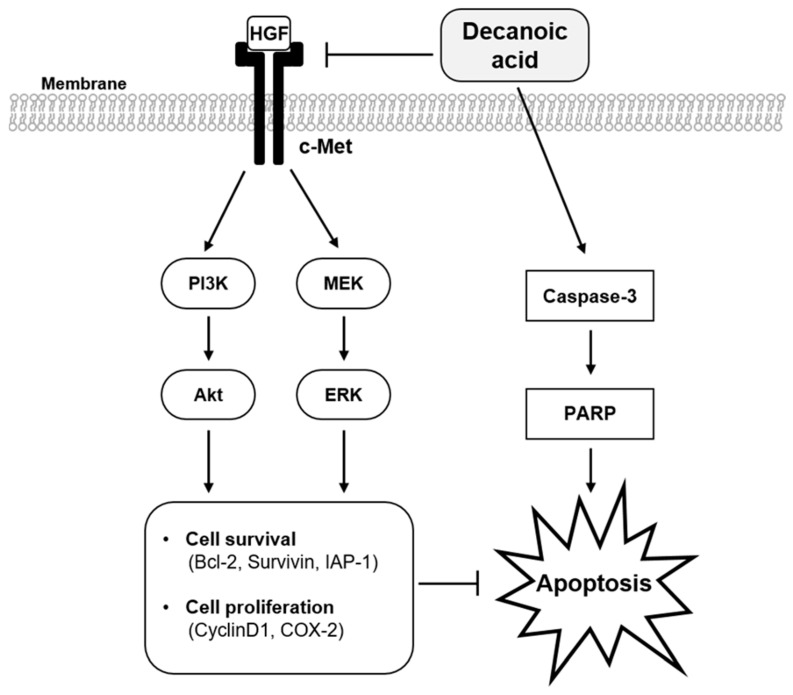
Schematic diagram of the effect of DA on c-Met signaling cascades.

Conventionally, the interaction between HGF/c-Met results in the phosphorylation of the tyrosine kinase domain at Y1234 and Y1235 residues. It may be noted that DA treatment displayed inhibitory activity towards phosphorylation of the tyrosine kinase domain. The other residues that can be phosphorylated are Y1349 and Y1356 at the multifunctional docking site domain and S985 and Y1003 at the juxtamembrane domain. The phosphorylation of Y1349 and Y1356 at the multifunctional docking site domain results in the recruitment of SH2 domain-containing proteins to the receptor complex and subsequent activation of downstream pathways such as PI3K/Akt/mTOR, MEK/ERK, and STAT3 signaling axes, which can promote tumorigenesis in diverse tumors, including HCC [10,14,17]. DA exerted inhibitory activity towards the phosphorylation of PI3K, Akt, MEK, and ERK, suggesting that all the c-Met-dependent pathways are downregulated following the suppression of c-Met activity. It is important to note that treatment of tumor cells with HGF resulted in an increase in phosphorylation of c-Met, PI3K, Akt, MEK, and ERK, whereas the inclusion of DA nullified the HGF-stimulated phosphorylation of these proteins. This data provided strong evidence that DA imparts its anticancer activity through modulating the c-Met signaling pathway, at least partly (Figure 6). It is important to note that PI3K/Akt/mTOR, MEK/ERK, and STAT3 transmit signals for the expression of genes that are associated with promotion of cell survival, antiapoptosis, proliferation, migration, invasion, metastasis, drug resistance, inflammation, and immune evasion [48,49,50]. Therefore, counteracting the effect of c-Met may curb the deleterious effects of all these oncogenic proteins in cancer. 

Since c-Met relays proliferative and survival signals for cancer cells, it was hypothesized that inhibition of c-Met must drive cell death. To verify this, HCC cells were treated with DA for the indicated time and dose and subjected to PI staining followed by flow cytometric analysis. DA piled up HCC cells in the sub-G1 stage, indicating that cells are losing viability. In a cell undergoing apoptosis, caspase-3 is activated, which results in the subsequent activation of caspase-dependent DNase (CAD). Functionally, CAD fragments the genomic DNA into oligomers, which are released out of the cell, and these cells have reduced DNA content (hence called hypodiploid cells) in comparison with cells in which apoptosis is not initiated. Hypodiploid cells appear as a sub-G1 population when stained with PI and subjected to flow cytometric analysis. Additionally, the apoptosis-inducing effect of DA was further confirmed using the annexin-V assay and the TUNEL assay. Activation of caspase-3 and PARP cleavage are the signature marks of cells committed to apoptosis. DA significantly induced the cleavage of caspase-3 and PARP in HCC cells. Proapoptotic signals from either caspase-8 or caspase-9 result in the activation of caspase-3. Subsequently, activated caspase acts on target proteins (such as focal adhesion kinase, lamins, protein kinase B, protein kinase C, actin, tubulin, gelsolin, CAD, etc.) with a preferred proteolytic cleavage sequence specificity of DEVDG (Asp-Glu-Val-Glu-Gly). In this study, HCC cells were exposed to a chemically modified peptide with a sequence of Z-DEVD-FMK, which serves as a potent inhibitor of caspase-3. The treatment of HCC cells with Z-DEVD-FMK resulted in the reversal of DA-triggered apoptosis. This assay further confirms that DA induces apoptosis, a form of death, in HCC cells. Bcl-2, Survivin, IAP-1, Cyclin D1, and COX-2 are some of the key proteins involved in opposing apoptosis, relaying survival signals, driving the cell cycle, and promoting inflammation in cancer cells. The role of these proteins in oncogenesis and tumor progression has been well demonstrated. All of these proteins are generally overexpressed in HCC cells, favoring the growth and proliferation of liver cancer cells. DA substantially downmodulated the expression of all these proteins in the tested cancer cell lines, indicating that DA interferes with the expression and functioning of these proteins to oppose the growth of HCC cells. All these assays were performed in the presence and absence of HGF, and DA consistently displayed apoptosis induction in the presence and absence of HGF. This observation may be inferred in a way that DA has the capability to abrogate constitutive as well as HGF-driven activation of c-Met and its downstream signaling events in HCC.

Next, the safety profile of DA was examined in the nude mice model. For this, DA (at doses of 5, 50, and 100 mg/kg) was administered intraperitoneally and examined for toxicity symptoms, including alteration in body weight, BUN, AST, and ALT. Experimental animals did not display a significant alteration in any of the tested parameters. The antitumor efficacy of DA was examined in the HCC orthotopic mice model following acute toxicity studies. The tumor growth and regression were analyzed by measuring the bioluminescence signals. A significant decline in tumor burden and lung metastasis was observed in the DA-treated group of animals in comparison with the vehicle-treated group, demonstrating that DA possesses good antitumor properties. The lung is the primary site of metastasis in HCC. Further, tumor tissues and lungs were subjected to immunohistochemical staining for the expression of phospho-c-Met, Ki-67, and CD31. There was a significant decline in the expression of all the tested proteins. The reduction in phospho-c-Met suggested that the constitutively active c-Met pathway is abrogated upon treatment with DA in tumor tissues. Ki-67 and CD31 are the biomarkers of tumors, and the reduction of their expression is an indication of a retardation of tumor proliferation. Pronounced inhibition of phosphorylation of c-Met, PI3K, Akt, MEK, and ERK was witnessed in the tumor tissues derived from DA-treated mice, inferring that the observed antitumor effects are primarily due to the mitigation of c-Met and its downstream oncogenic pathways. Taken together, DA was found to impart growth-inhibitory effects in HCC cells and tissues by abrogating c-Met and associated oncogenic signaling events.

## 4. Materials and Methods

### 4.1. Reagents

Decanoic acid (purity ≥ 98.0%) was purchased from Sigma-Aldrich (St. Louis, MO, USA). HGF was obtained from PeproTech (Offenbach, Germany). Antibodies of Cyclin D1, β-actin, IAP-1, Akt, PARP, Bcl-2, COX-2, and Survivin were obtained from Santa Cruz Biotechnology (Santa Cruz, CA, USA). Antibodies of ERK, MEK, PI3K, c-Met, p-ERK, p-MEK, p-AKT, p-PI3K, and cleaved caspase-3 were procured from Cell Signaling Technology (Beverly, MA, USA). 

### 4.2. Cell Culture Conditions

Information about the procurement of HCC cell lines has been described in our previous study [51].

### 4.3. MTT Assay

HCCLM3 and HepG2 cells were pre-treated with DA (0, 20, 40, 60, and 80 μM) for 2 h and then exposed to HGF (50 ng/mL) for a total of 48 h. The viability of cells was determined through the MTT assay, as described earlier [52]. In brief, the cells were grown in a 96-well plate and incubated with DA for the indicated time at the indicated concentrations. Thereafter, MTT solution (20 µL; 5mg/mL) was added to each well and allowed to stand for 2 h. Purple-colored crystals are formed in each well, depending on the number of viable cells. Subsequently, the medium is carefully removed, followed by the addition of lysis buffer (0.1 mL; 20% SDS, 50% dimethylformamide). The lysis buffer dissolves the purple crystals, whose optical density is measured at 570 nm using a plate reader.

### 4.4. Immunocytochemistry

HCC cells were exposed to DA (80 μM) for 2 h and treated with HGF (50 ng/mL) for 30 min. They were fixed with 4% paraformaldehyde for 20 min and incubated with 0.2% Triton-X-100 for 10 min. Thereafter, immunocytochemistry was performed as reported earlier [53].

### 4.5. Cell Cycle Analysis

The cells were treated with DA and HGF, and cell cycle analysis was performed as described earlier [53]. DA and HGF-exposed cells were harvested by trypsinization, washed with PBS, and fixed using 70% ethyl alcohol. Before the analysis, cells were treated with RNAse A for 30 min and incubated with propidium iodide (PI) for 30 min at room temperature. Subsequently, these cells were analyzed for cell cycle distribution using flow cytometry.

### 4.6. Annexin V Assay

HCCLM3 and HepG2 cells were treated with DA for 24 h, an annexin V assay was performed as reported before [52].

### 4.7. TUNEL Assay

HCC cells were treated with DA or HGF at the indicated concentrations and times, and thereafter, the TUNEL assay was performed as described before [52].

### 4.8. Acute Toxicity Studies

In vivo experiments were conducted based on protocols approved by the SingHealth Institutional Animal Use and Care Committee. For toxicity analysis, eight-week-old NCr nude female mice were administered intraperitoneal injections of 5 mg/kg, 50 mg/kg, and 100mg/kg of DA and vehicle (0.1% DMSO). Thereafter, acute toxicity was evaluated as described earlier [51].

### 4.9. In Vivo Experiments

Eight-week-NCr nude female mice were implanted with HCCLM3-Luc cells orthotopically. When the bioluminescence signal reached 10^6^, mice were either treated intraperitoneally with vehicle (0.1% DMSO) or DA (50 mg/kg), thrice a week, for four weeks. They were then euthanized and liver as well as lung tissues were obtained, and stored at −80 °C.

### 4.10. Immunohistochemistry (IHC)

The tumors obtained from the vehicle and treatment groups of mice were fixed with neutral buffered formalin (10%, BBC Biochemical, Mount Vernon, WA, USA). They were processed and made into paraffin blocks. Then, IHC was performed as described earlier [54]. 

### 4.11. Statistical Analysis

The results are expressed as means ± standard deviation (SD), and one-way ANOVA with the Benferroni multiple comparison test or unpaired *t*-test with Welch’s correction was done (GraphPad Prism 5.0; GraphPad Software, San Diego, CA, USA). A *p*-value of 0.05 or less was considered significant.

## 5. Conclusions

In conclusion, our findings suggest that DA imparts potential anti-cancer effects by targeting the c-Met pathway and other c-Met-dependent oncogenic signaling events that are associated with hepatocarcinogenesis. DA was found to induce apoptosis in HGF-induced and uninduced HCC cells. The observed in vitro anticancer effects were supported by the results of the in vivo studies performed using a preclinical HCC mice model. These findings can be promising for developing effective c-Met inhibitors from natural agents, although their long-term safety and bioavailability still need to be evaluated in future studies. This study supports the idea that DA has the potential to be examined in a clinical setting for its antitumor activity in c-Met-positive tumors. 

## Data Availability

The data presented in this study are available upon request from the corresponding authors.

## Supplementary Materials

The following supporting information can be downloaded at: https://www.mdpi.com/article/10.3390/cancers15194681/s1, File S1: Western blot information.

## Abbreviations

HCC	hepatocellular carcinoma
DA	decanoic acid
HGF	hepatocyte growth factor
RTK	receptor tyrosine kinase
NT	non-treated
FBS	fetal bovine serum
IHC	immunohistochemistry
