# Supplementary figures and images for: Decanoic Acid Exerts Its Anti-Tumor Effects via Targeting c-Met Signaling Cascades in Hepatocellular Carcinoma Model
Source: Cancers (Basel). 2023 Sep 22;15(19):4681. doi: 10.3390/cancers15194681 (PMC10571573; doi:10.3390/cancers15194681)

Fig.1D

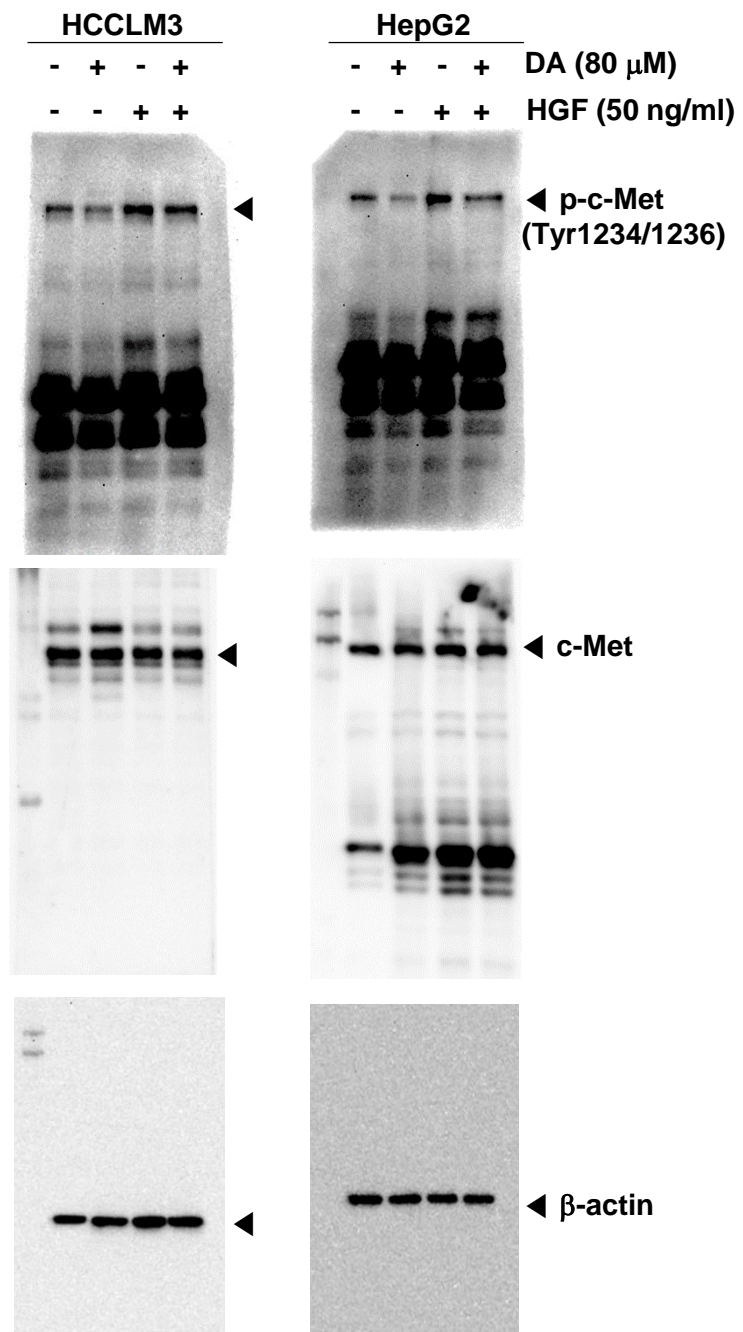

Fig.1E

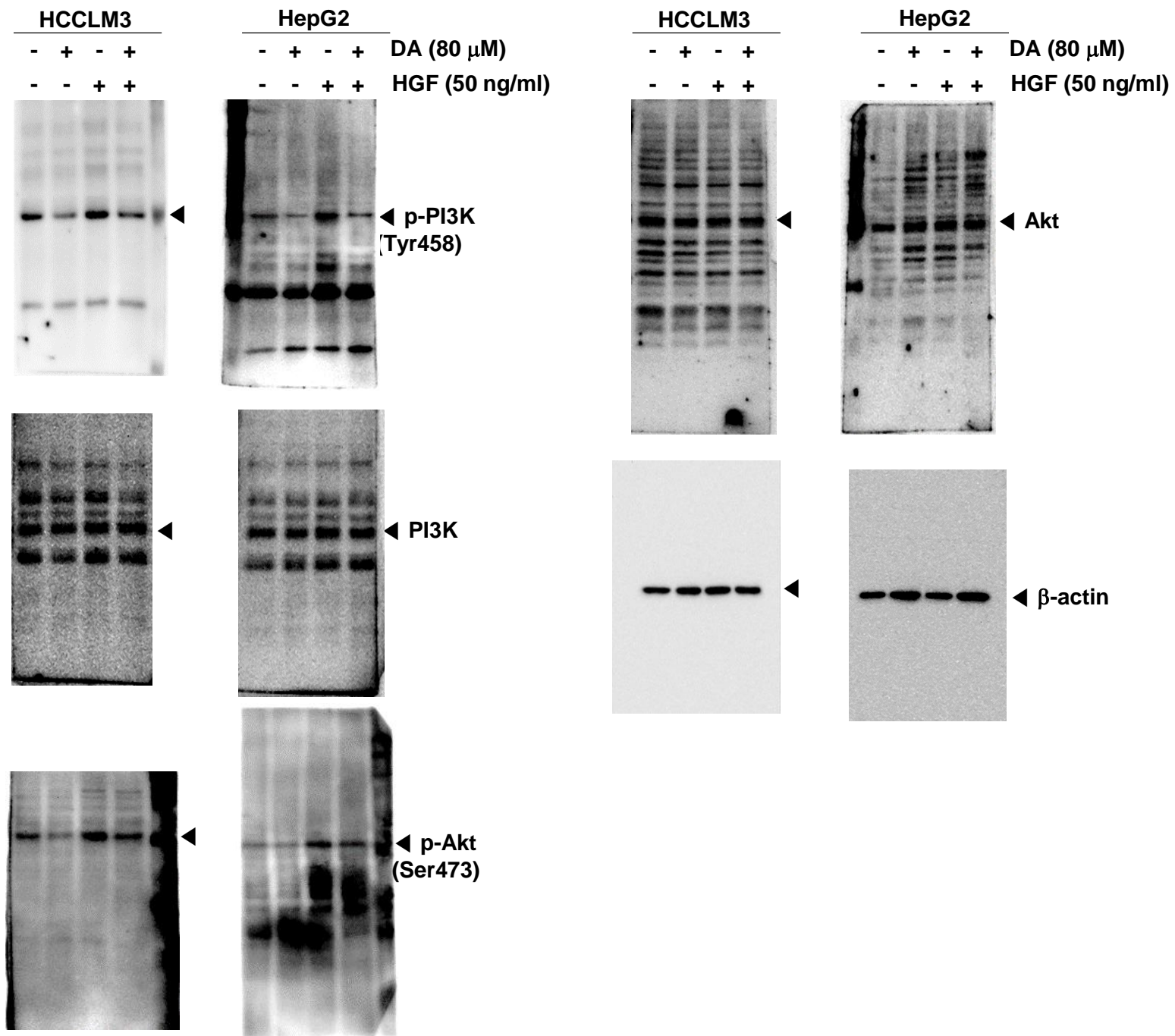

**Fig.1F**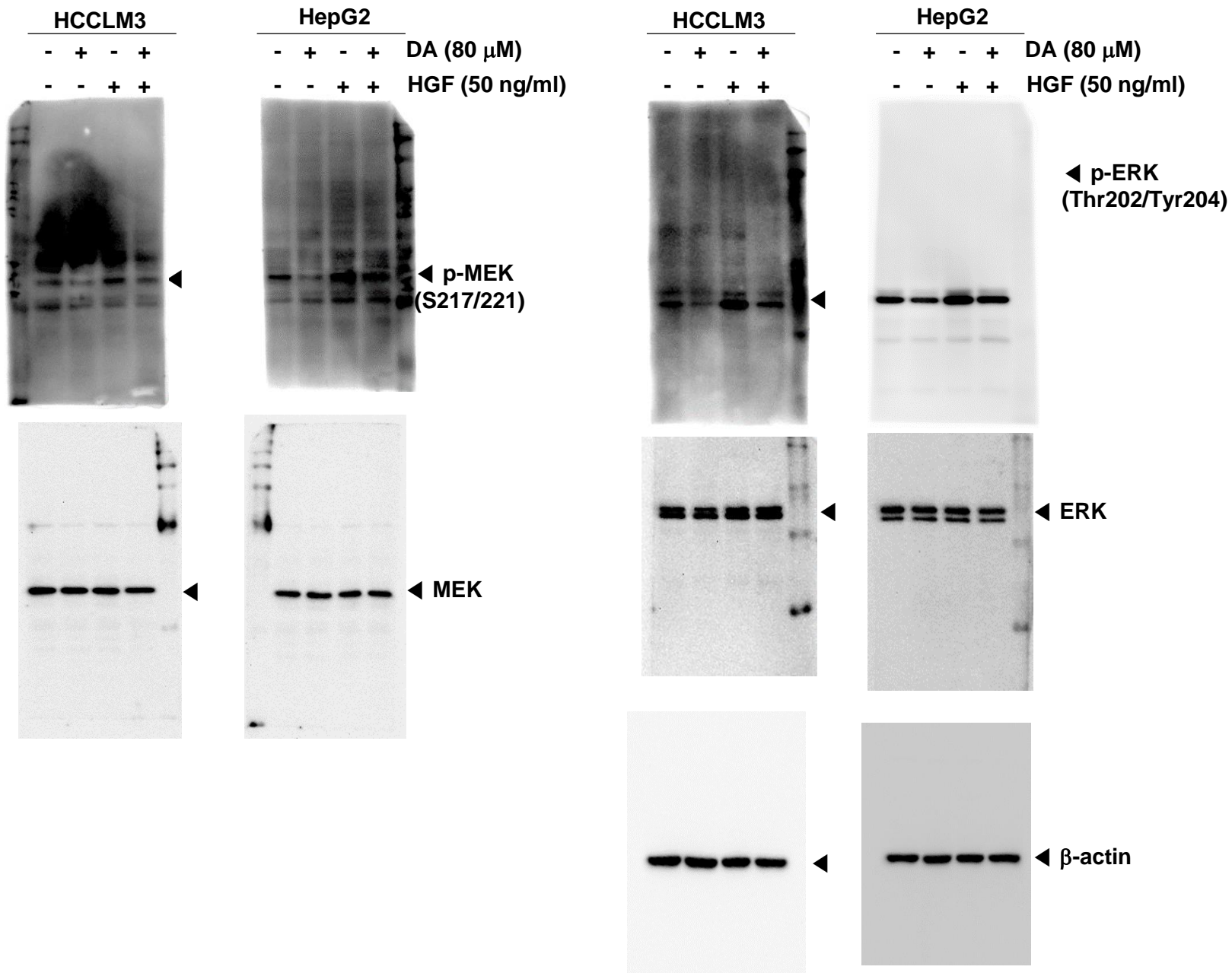

**Fig.2D**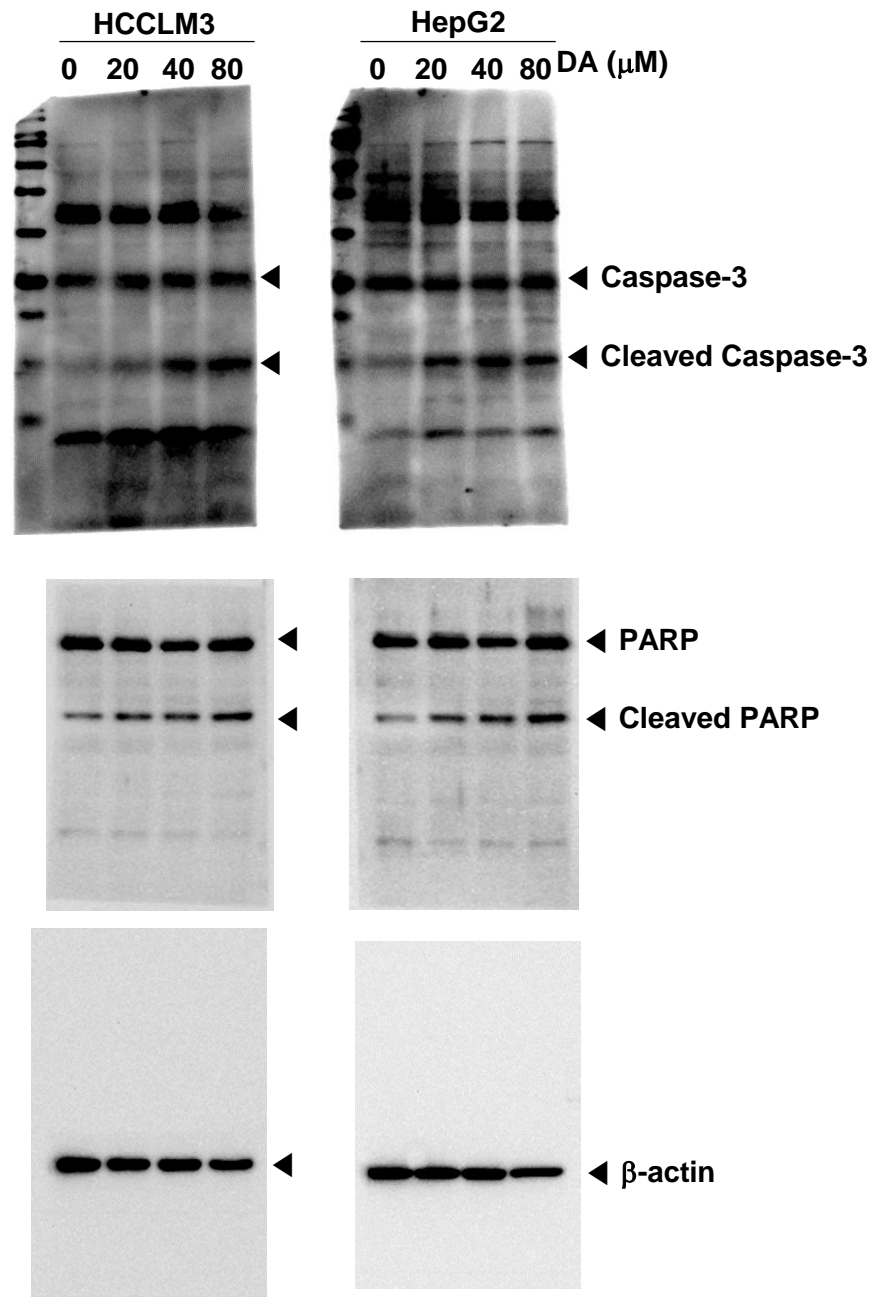

Fig.2E

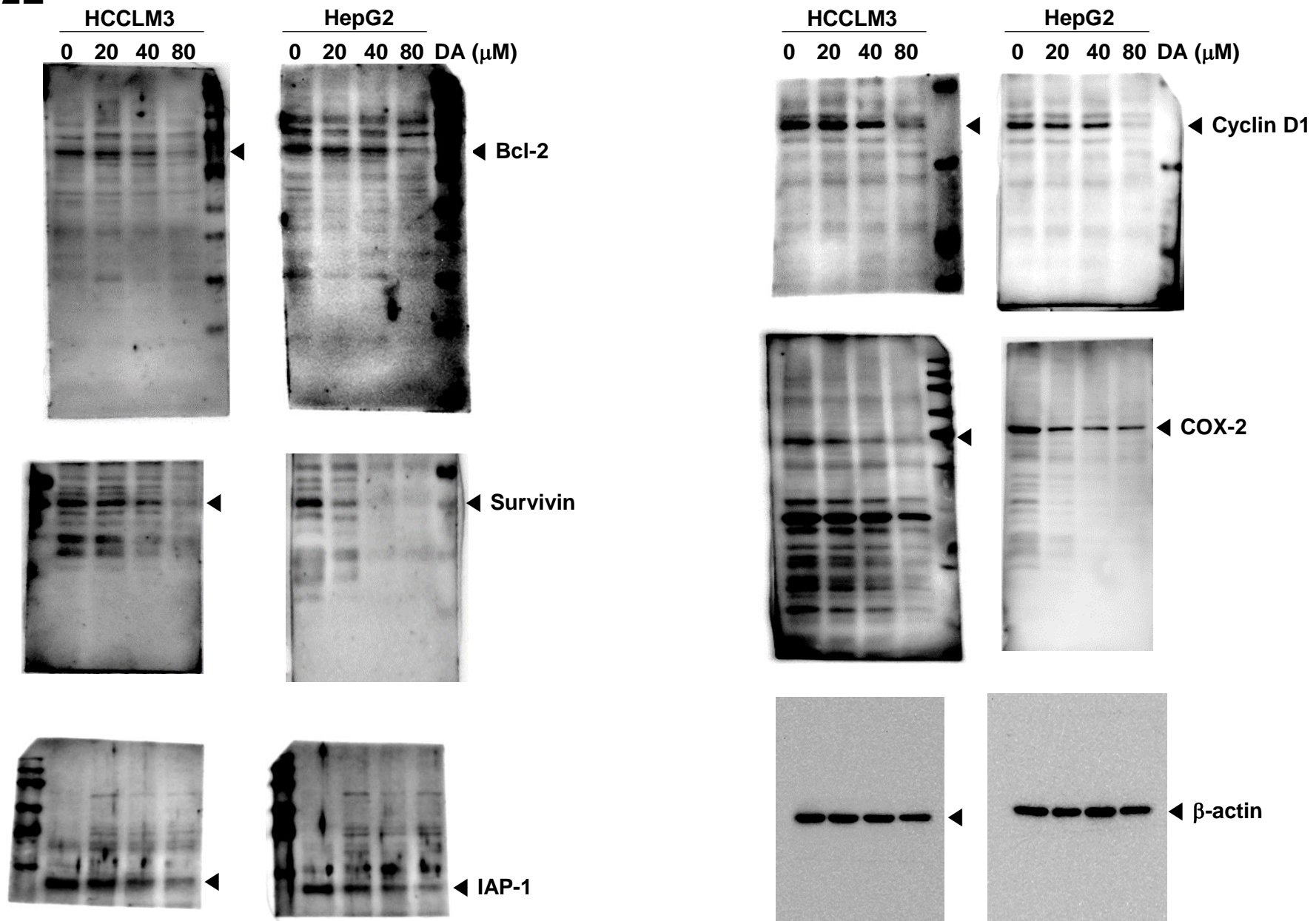

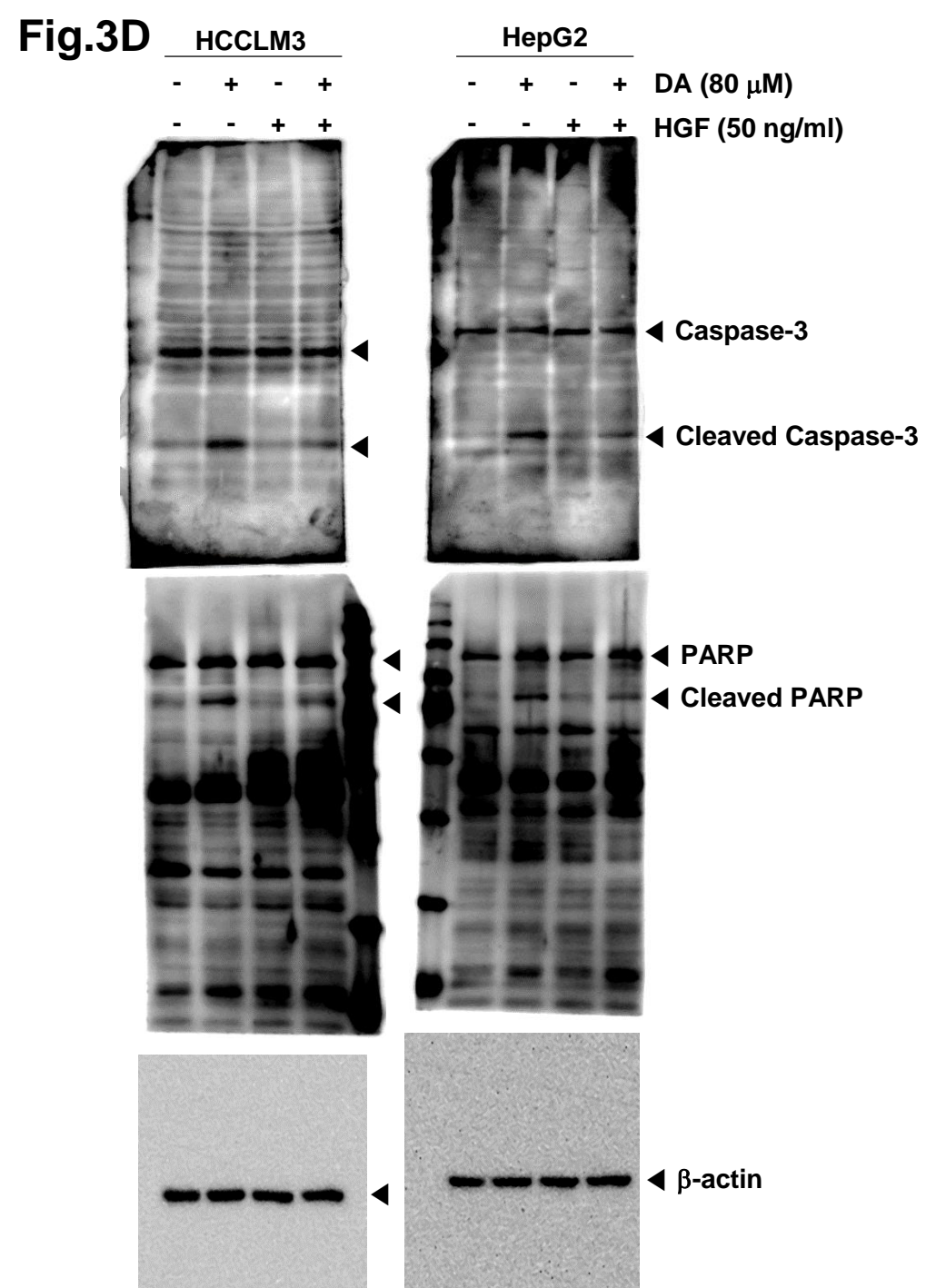

Fig.3E

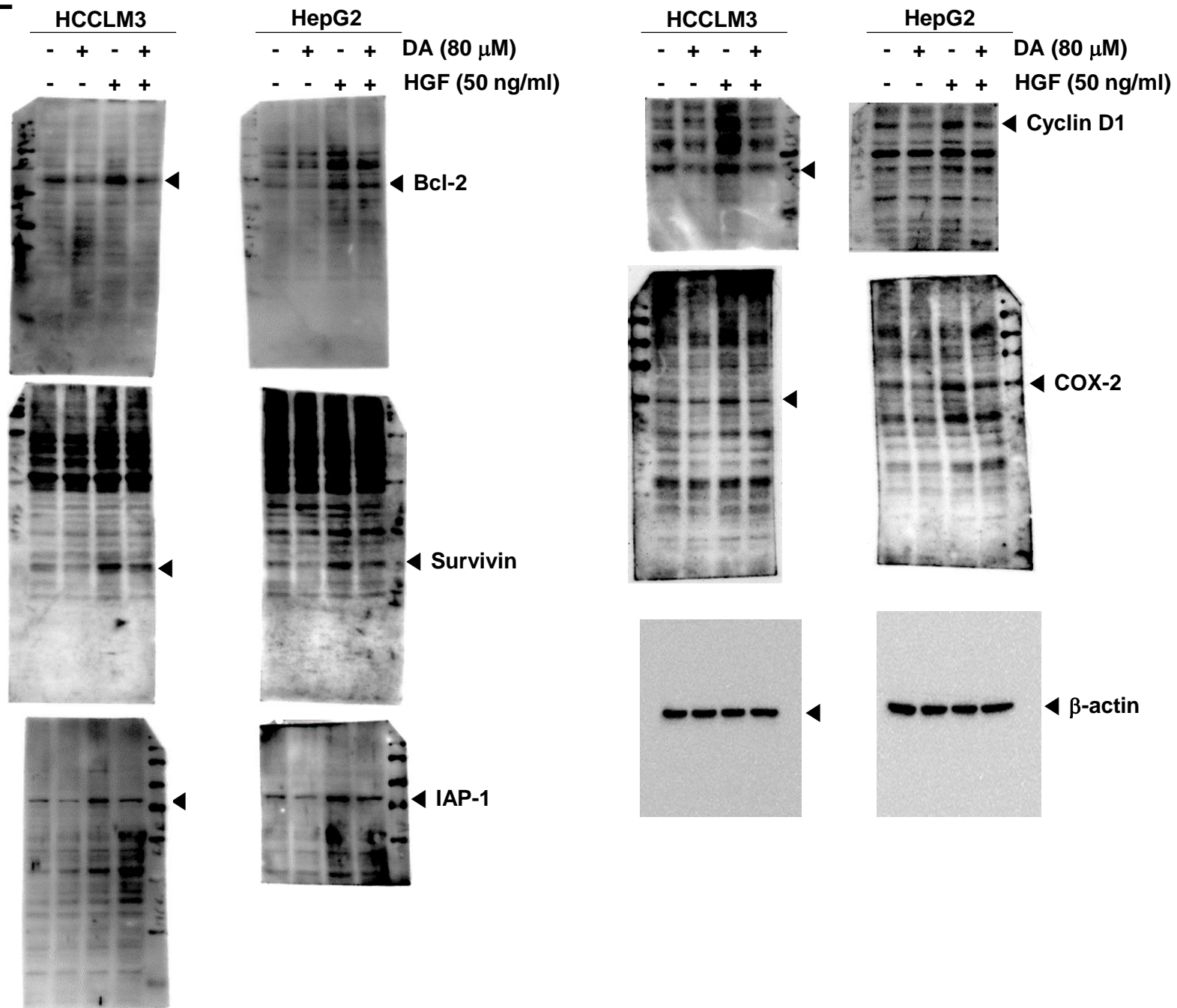

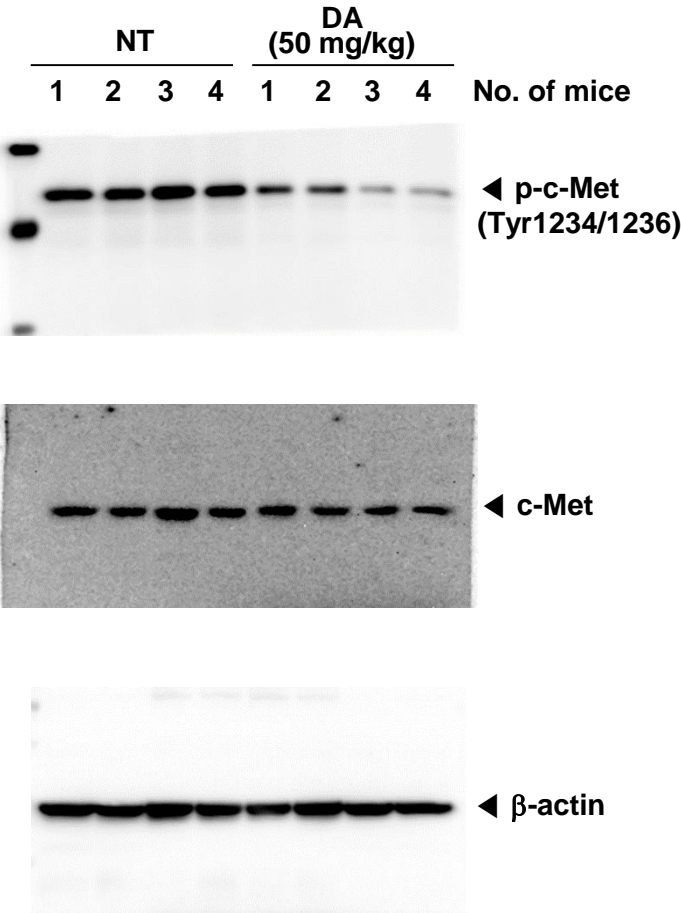

**Fig.5C**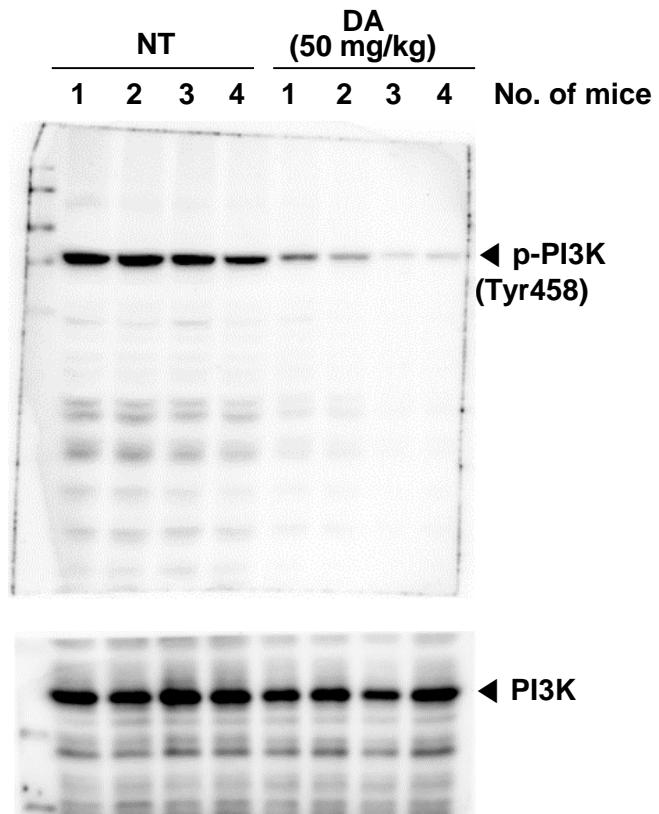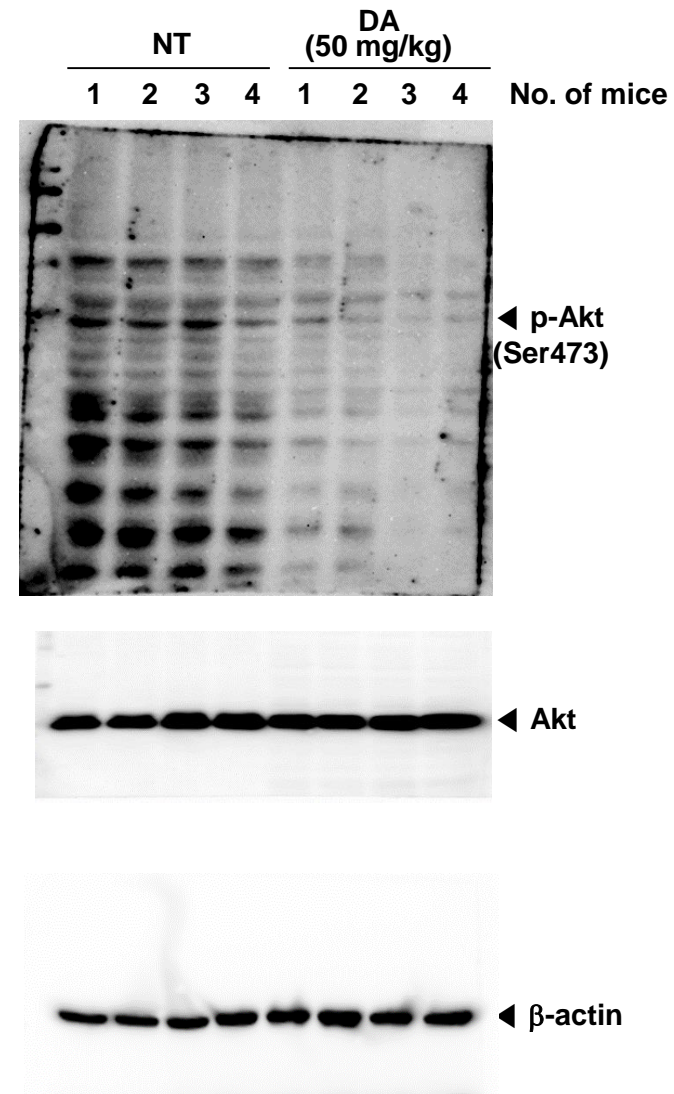

Fig.5D

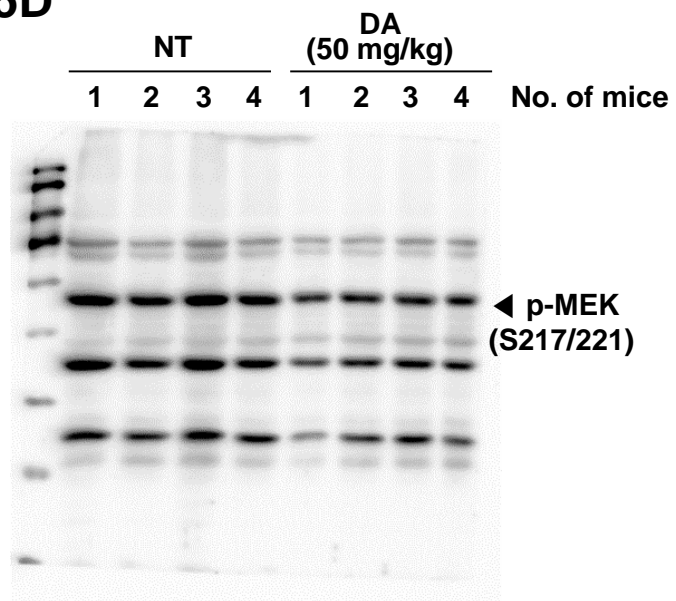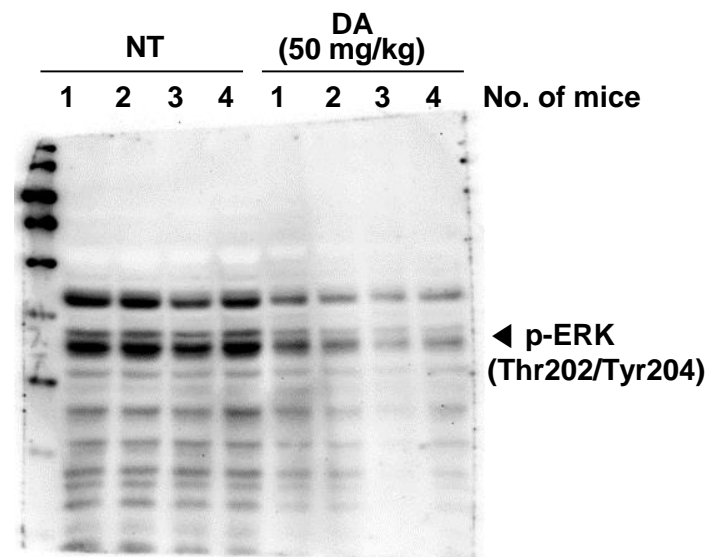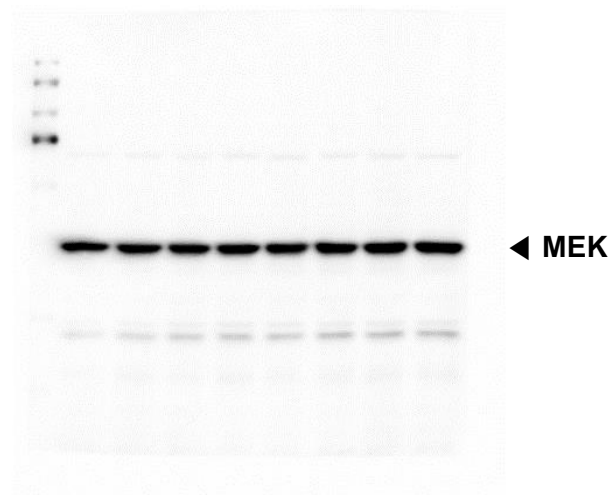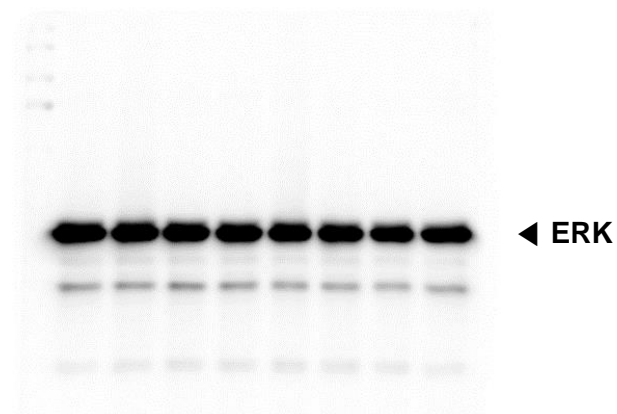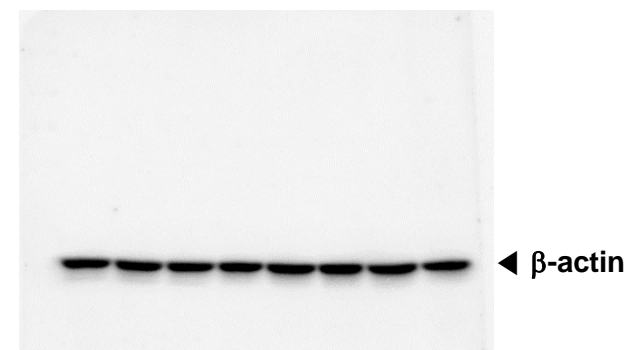

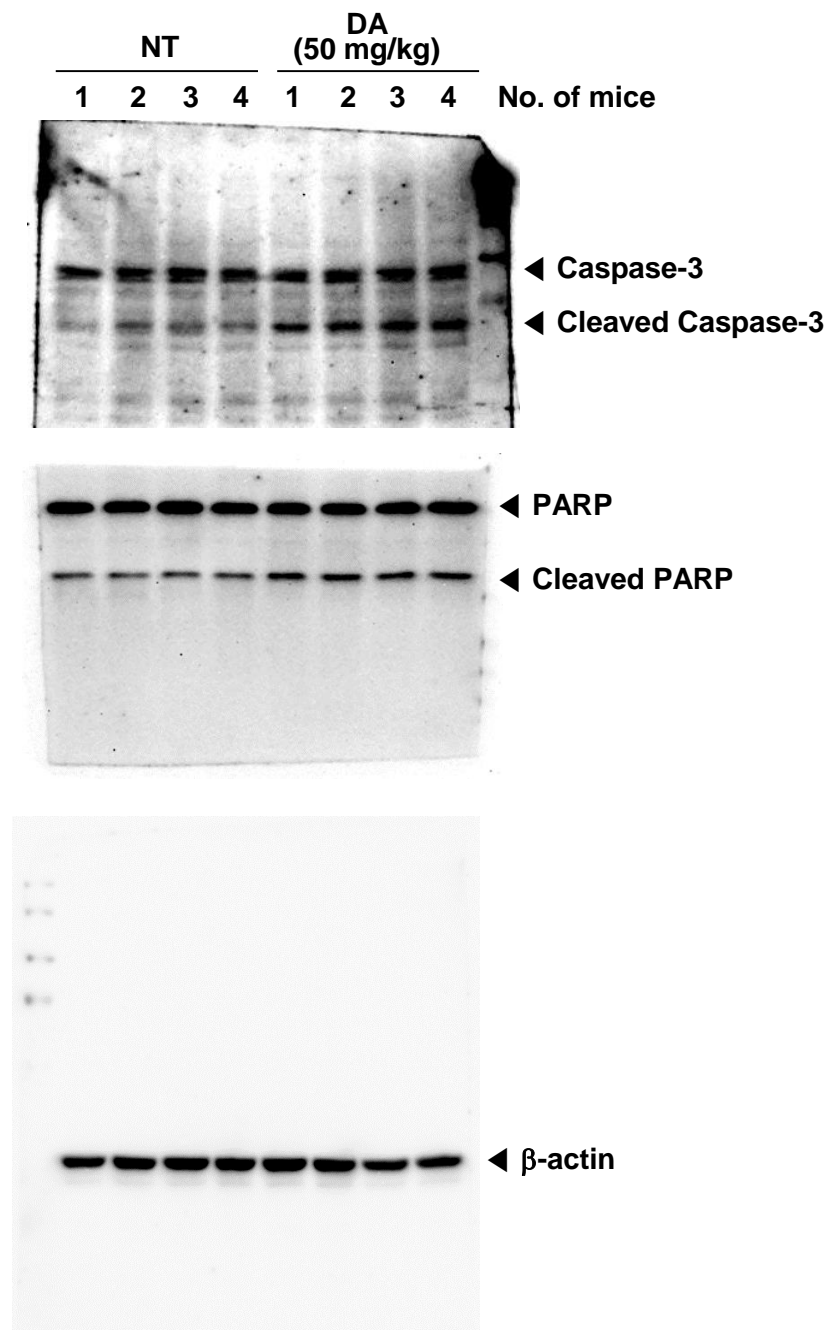

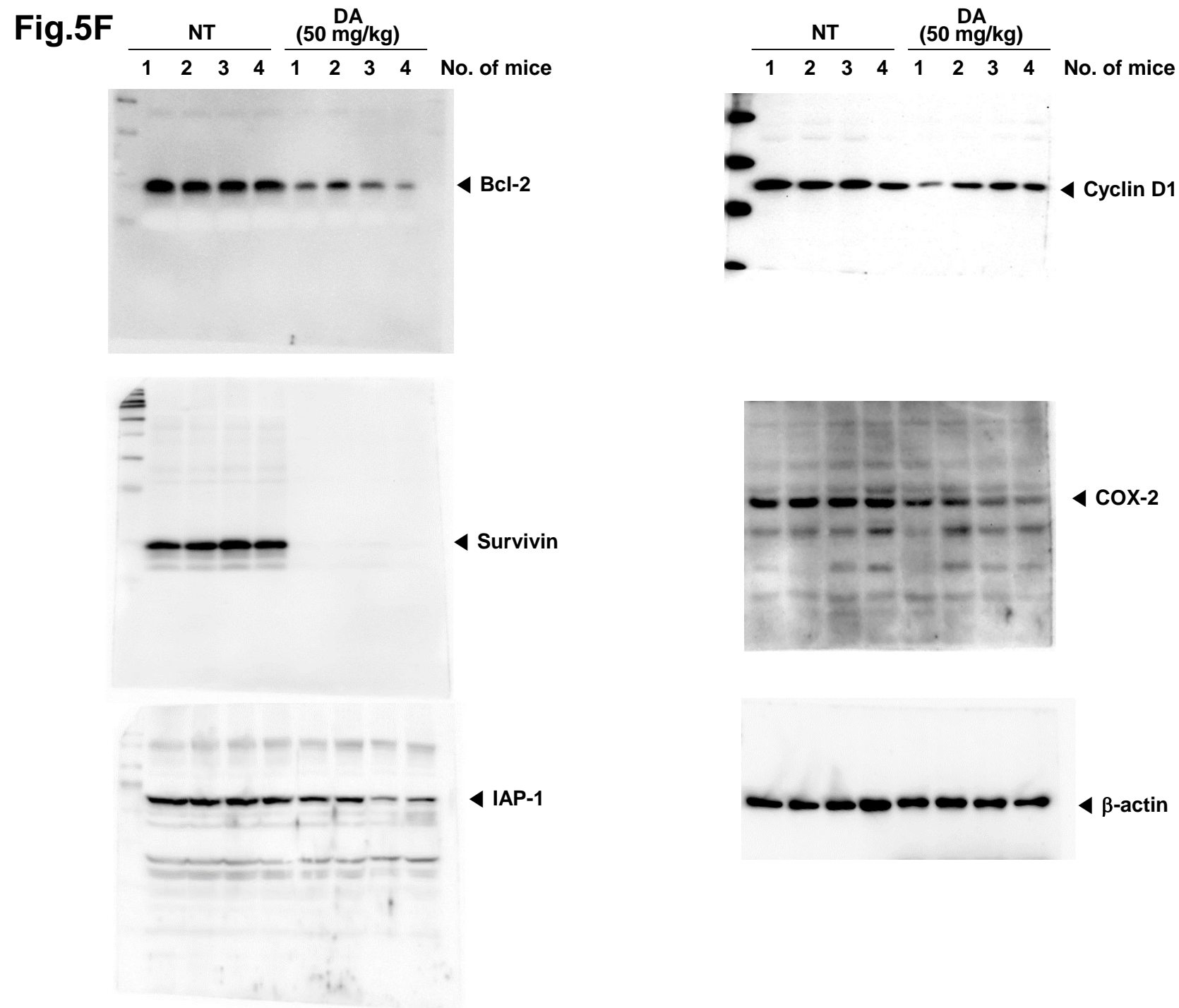

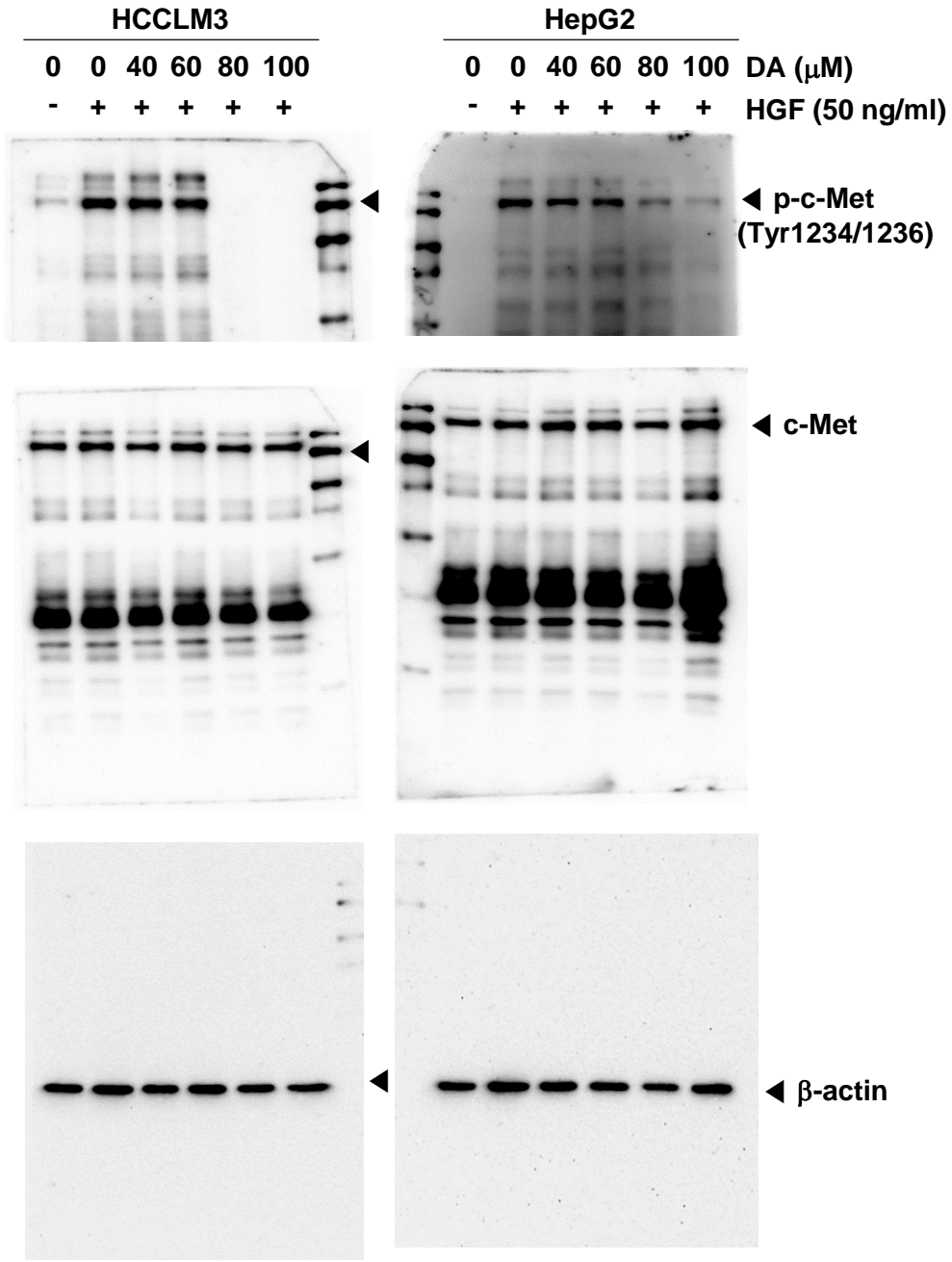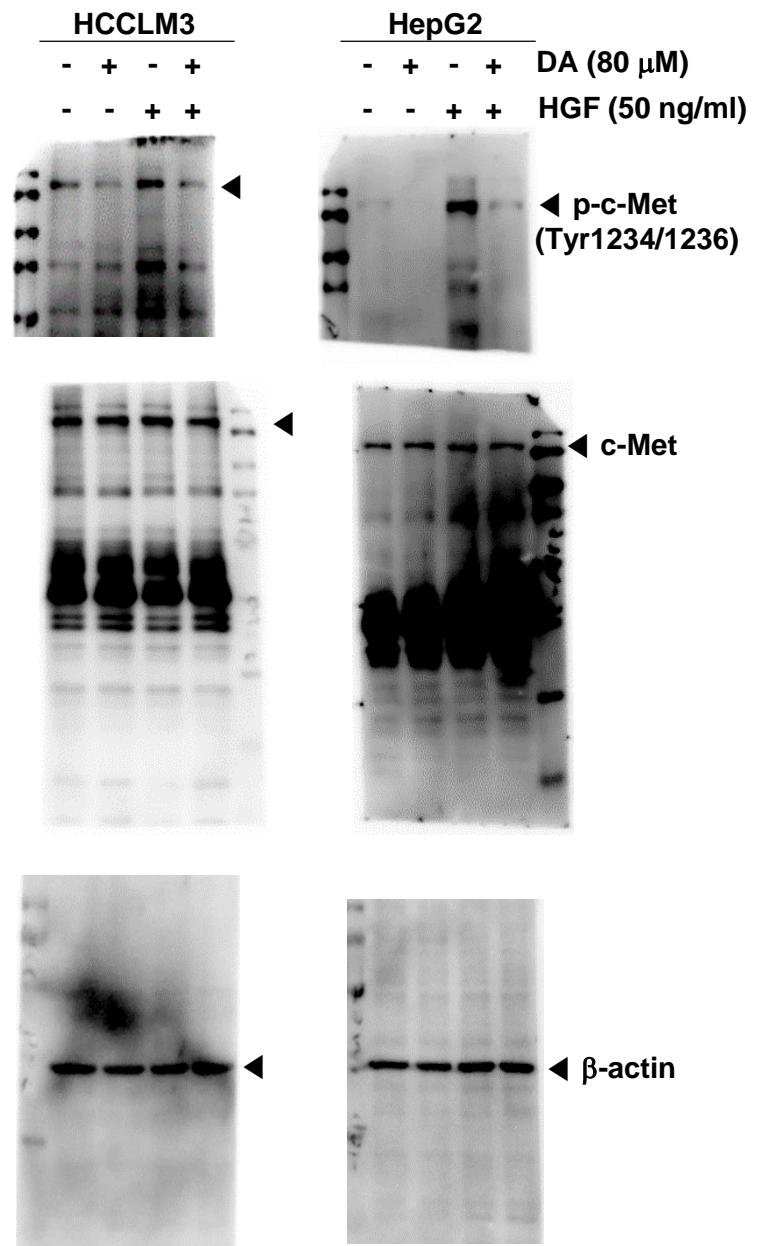

Supplement: Supplementary file 1 [file cancers-15-04681-s001.zip › cancers-2551223-supplementary.pdf]
